# Unraveling the Molecular Mechanisms Linking Cigarette Smoke Exposure to Skin Damage

**DOI:** 10.3390/ijms27052392

**Published:** 2026-03-04

**Authors:** Ziyi Tan, Yuping Wei, Shengan Zhang, Jianping Song, Wei Zhu

**Affiliations:** 1 Artemisinin Research Center, Guangzhou University of Chinese Medicine, Guangzhou 510120, China; tziyi1021@163.com (Z.T.);; 2 School of Traditional Chinese Medicine, Guangdong Pharmaceutical University, Guangzhou 510006, China; 3 The Second Clinical School of Medicine, Guangzhou University of Chinese Medicine, Guangzhou 510006, China

**Keywords:** cybertoxicology, molecular docking, cigarette smoke, skin damage

## Abstract

CS is an environmental pollutant everywhere, but we still do not fully know how it hurts our skin. This study integrates LC-MS, network toxicology, molecular docking, and experimental validation in order to understand how CS causes skin involvement at the molecular level. By searching a database, constructing a PPI network and analyzing GO/KEGG, we found 57 candidate targets related to CS-induced skin damage. We found that STAT3, AKT1, TP53, CASP3 and IL-6 play the core roles, and PI3K-Akt, p53, JAK-STAT and apoptosis pathways may be crucial. Molecular docking analysis confirmed strong interactions between components of CS and these key targets. In vitro validation using HaCaT cells showed that CS exposure decreased expressions of STAT3 and AKT, but increased p53, CASP3 and IL-6. The inhibition of PI3K-AKT- and JAK-STAT-related responses, coupled with the initiation of p53-driven apoptosis, led to the observed cytotoxicity, functional impairment, oxidative stress and inflammation, which induced and aggravated skin damage. These findings provide a new perspective on the harmful effects of CS on the skin, providing both a theoretical basis for strengthening regulatory measures to limit exposure and opening new avenues for exploring relevant prevention strategies.

## 1. Introduction

Cigarette smoke (CS) is a common and complex environmental pollutant containing more than 7000 compounds produced during combustion. These compounds include known toxic components, such as nicotine, polycyclic aromatic hydrocarbons (PAHs), tobacco-specific nitrosamines (TSNAs) and formaldehyde [1,2,3]. Despite the widespread recognition of its harm by public health institutions around the world and the implementation of tobacco control policies, the smoking rate remains high. According to a report by the World Health Organization (WHO) in 2024, there are about 1.25 billion smokers worldwide, which shows that smoking is still a serious challenge facing global public health [4].

One of the body’s largest organs, the skin, serves as the primary interface with the external environment [5]. It is the first physical and immune barrier to resist environmental aggression. More and more evidence shows that CS has a clear toxic effect on skin tissue and may induce or aggravate a variety of skin diseases. Multiple studies have observed an association between CS exposure and impaired wound healing, premature skin aging, psoriasis, acne, eczema, hidradenitis suppurativa, hair loss, squamous cell carcinoma and melanoma [1,6,7]. In addition, CS will also aggravate the skin condition of patients with diabetes, HIV/AIDS and lupus erythematosus [6]. At the physiological and pathological levels, smokers show reduced skin blood flow and impaired local immune function, resulting in chronic microcirculatory damage and increased risk of skin infection [8,9,10]. The key components of CS, such as nicotine, have been shown to inhibit the activity, migration, proliferation and remodeling ability of dermal fibroblasts, thus hindering wound repair [11]. Moreover, epidemiological data link CS contact with early skin aging. Compared with non-smokers, the facial aging and skin wrinkles of smokers are more obvious. It is estimated that smoking 20 cigarettes a day will accelerate the inherent aging of the skin for nearly ten years [12,13].

Yet existing studies on CS’s harmful health impacts primarily focus on respiratory, cardiovascular, and carcinogenic consequences. In contrast, although its clear skin toxicity has been recognized, there are still relatively few studies on the molecular mechanism of skin damage induced by CS, and there is a lack of systematic clarification. With the rapid development of bioinformatics, network toxicology has become a powerful tool for evaluating the biological effects and mechanisms of environmental pollutants and efficiently and comprehensively predicting toxic pathways and core targets.

In order to study the molecular secrets behind the skin damage caused by CS, we used network toxicology, molecular docking and experimental verification. We first screened a database for potential targets associated with CS active ingredients and skin damage. Then, we built a protein–protein interaction (PPI) network to find out the key roles. We also performed Gene Ontology (GO) functional enrichment analysis and Kyoto Encyclopedia of Genes and Genomes (KEGG) pathway analysis to clarify related biological processes and signal transduction. In addition, we also calculated the binding capacity between the found CS compounds and core targets by molecular docking simulation. In order to simulate real-world cutaneous exposure to CS, we treated human skin cells HaCaT with cigarette smoke extract (CSE) and then conducted in vitro toxicity experiments to confirm the functions of core targets and pathways. This integrated approach primarily aims to delineate toxicological processes and identify potential therapeutic targets, thereby establishing a scientific foundation for early intervention against CS-induced skin damage.

## 2. Results

### 2.1. Chemical Composition of CSE

To analyze environmental pollutants in CSE, we used the UltiMate 3000 UPLC+-Q EXACTIVE Focus LC-MS system. Figure S1a,b display Total Ion Chromatograms (TICs) under positive and negative ion conditions, respectively. The composition of environmental pollutants identified shows that alkaloids and PAHs are the main components. Among the 10 alkaloids and PAHs with the highest content in CSE (Table 1), nicotine has the highest content in alkaloids, and Phenanthrene has the highest content in PAHs.

### 2.2. Network Toxicology Analysis

#### 2.2.1. Skin Toxicity Assessment of CS Components

Using three prediction platforms, namely, AttentionSkin, ADMETLAB 3.0 and Pred-Skin 3.0, the skin toxicity of the above 20 components was evaluated. Compounds predicted to be irritating, corrosive or sensitizing are classified as skin toxicological hazards at different degrees (Table 2). The evaluation results are combined with toxicological information from PubChem (https://pubchem.ncbi.nlm.nih.gov, accessed on 21 August 2025) [14]. The results show that all 20 ingredients exhibit some degree of skin toxicity. Notably, two components, Myosmine and 2-methyltryptoline, demonstrated multiple hazards across all three prediction platforms, including skin irritation, corrosion potential, and sensitization risk, suggesting they are high-risk substances.

#### 2.2.2. Potential Targets of CS-Induced Skin Damage

**Figure 1:**
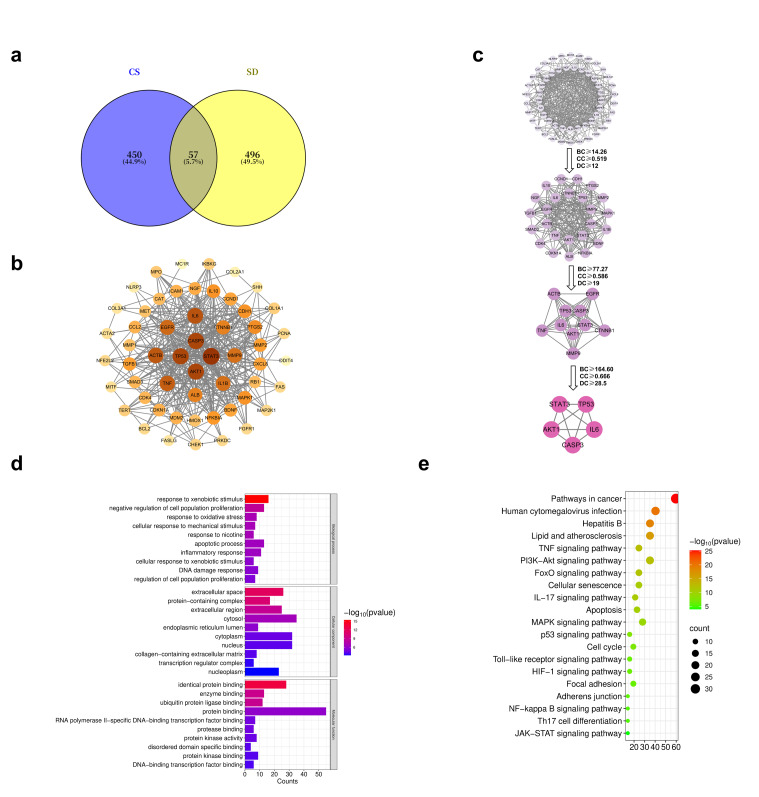
Study on network toxicology of skin damage caused by CS. (**a**). Venn diagram illustrating the overlap between predicted targets of cigarette smoke-related compounds (CS) and skin damage-associated targets (SD). The numbers within each circle indicate the total number of targets identified in each dataset, while the overlapping region represents the shared targets used for subsequent analysis. (**b**). PPI network of potential targets involved in CS-induced skin damage. The color and size of the nodes correspond to their degree values. (**c**). The core target of CS-induced skin damage. The nodes were filtered sequentially based on three centrality parameters: degree centrality (DC), betweenness centrality (BC), and closeness centrality (CC). The arrows indicate the progressive screening process using increasingly stringent thresholds to identify core hub targets. (**d**). GO enrichment analysis of CS-induced skin damage, including biological process (BP), cellular component (CC), and molecular function (MF) categories. The color gradient indicates statistical significance (–log_10_ *p* value). (**e**). KEGG pathway of skin damage caused by CS. The size of each dot represents the number of genes enriched in the pathway, and the color gradient indicates statistical significance (−log_10_ *p* value).

Analysis of the 20 CS components using the Swiss Target Prediction, SEA, and CTD databases yielded the following number of targets per component: nicotine (161), Norharman (63), Harman (76), Myosmine (27), Cotinine (48), Harmaline (38), 2-methyltryptoline (114), Harmalol (18), Nornicotine (28), Harmine (116), Phenanthrene (35), 2-Naphthylamine (19), Fluorene (14), Naphthalene (32), 1-Naphthylamine (30), 9-Fluorenone (55), 1-hydroxypyrene (32), Chrysene (22), 9-Acetylphenanthrene (38), Benzanthrone (43). A total of 507 CS-related targets were obtained (Table S1). Using the GeneCards, TTD, and OMIM databases, 553 skin damage-related targets were acquired (Table S1). Fifty-seven overlapping targets were identified as potential targets for CS-induced skin damage (Table S1, Figure 1a).

#### 2.2.3. Key Targets and PPI Network Construction for CS-Induced Skin Damage

The 57 candidate targets for CS-induced skin damage were entered into STRING to establish a PPI network. As visualized using Cytoscape (v3.10.0), removing two discrete nodes revealed a network containing 55 nodes and 373 edges (Figure 1b). Using the CytoNCA plugin, three consecutive screenings selecting nodes with values greater than or equal to the median for BC, CC, and DC identified five key targets: STAT3, AKT1, TP53, CASP3, and IL-6 (Figure 1c).

#### 2.2.4. GO/KEGG Enrichment Analysis

We used DAVID to analyze the GO/KEGG enrichment of the 57 targets. GO functional analysis highlighted biological processes associated with apoptosis, cell proliferation, inflammation, and oxidative stress responses. Cellular components were mainly associated with the extracellular matrix, the nucleus, and the cytoplasm. Molecular functions were primarily involved in signal transduction, protein interaction, and enzyme activity regulation (Figure 1d). Through the analysis of pathway enrichment by KEGG, it was found that the skin damage caused by CS is mainly related to some pathways, including carcinogenesis, viral interactions, PI3K-Akt signaling, p53 signaling, JAK-STAT signaling, and apoptosis (Figure 1e).

### 2.3. Cell Experiments

#### 2.3.1. CS Exposure Increases LDH Release in HaCaT Cells

**Figure 2:**
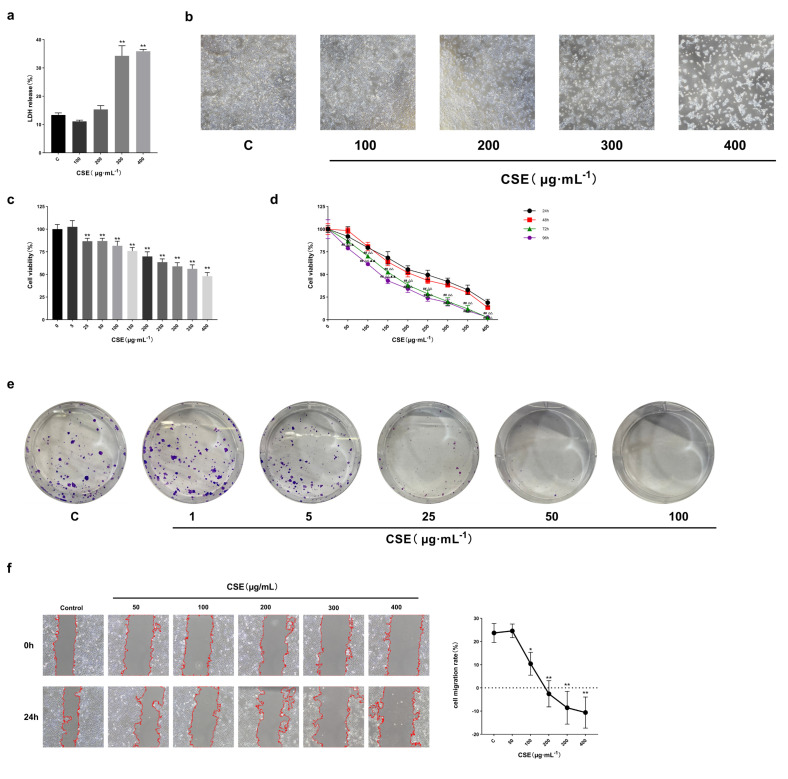
(**a**). Effect of different CSE concentrations after 24 h on cellular LDH release, *n* = 3/group. (**b**). Changes in cell morphology after 24 h of CSE with different concentrations. (**c**). The results of cell metabolic activity after 24 h of exposure to different concentrations of CSE, *n* = 5/group. (**d**). Effects of different CSE concentrations and exposure time on cell metabolic activity, *n* = 5/group. (**e**). Clonogenic assay assessing long-term proliferative capacity of HaCaT cells following CSE exposure. (**f**). Effects of different concentrations of CSE on cell wound healing response after 24 h, *n* = 3/group. All data are displayed as mean ± SD. * *p* < 0.05, ** *p* < 0.01 (vs. control group); ## *p* < 0.01 (vs. 24-h group); △△ *p* < 0.01 (vs. 48-h group); ▲ *p* < 0.05, ▲▲ *p* < 0.01 (vs. 72-h group).

LDH release is considered an indicator of cytotoxicity. After exposing HaCaT cells to different concentrations of CSE (100, 200, 300, 400 µg/mL) for 24 h, LDH release was measured, and cell morphology was observed. The results show that LDH release increased significantly at 300 µg/mL CSE compared to the control group and further increased with higher CSE concentrations (Figure 2a). Morphological changes were also observed with increasing CSE concentration, including widened intercellular spaces, loose cell arrangement, vacuolated and shrunken cells, and floating, highly refractive dead cells (Figure 2b).

#### 2.3.2. CS Exposure Impairs Metabolic Activity, Wound Healing Response, and Proliferative Capacity of HaCaT Cells

We determined the toxic effects of CS on skin, considering the findings from the core targets and GO/KEGG analysis that suggest CS affects cell activity, proliferation, and migration. HaCaT cells were exposed to various CSE concentrations (0, 5, 25, 50, 100, 150, 200, 250, 300, 350, 400 µg/mL) for 24 h and for different durations (24, 48, 72, 96 h). The MTT assay findings reveal that CS significantly affects HaCaT cell metabolic activity depending on the dose and exposure time relative to the control group (Figure 2c,d). It should be noted that MTT measurements reflect mitochondrial reducing capacity and therefore represent cellular metabolic activity rather than absolute cell viability, particularly under conditions of severe cytotoxic stress. As shown in Figure 2e, colony formation of HaCaT cells was markedly suppressed by CSE in a concentration-dependent manner. Notably, CSE concentrations ≥ 50 μg/mL almost completely abolished colony formation, indicating a profound impairment of long-term proliferative capacity, even at doses that did not induce overt acute cytotoxicity within 24 h. In the wound healing assay, CSE exposure resulted in a dose-dependent delay in scratch closure (Figure 2f). At lower concentrations (≤100 μg/mL), HaCaT cells exhibited a partial but measurable reduction in wound closure compared with control cells. In contrast, exposure to 300 μg/mL CSE almost completely abolished scratch closure over the observation period. Notably, this pronounced inhibition occurred under conditions associated with markedly reduced cell metabolic activity and proliferative capacity, indicating that impaired wound closure at high CSE concentrations reflects a combined effect of reduced cell viability, compromised proliferative potential, and altered cell motile behavior, rather than an isolated defect in migration alone.

#### 2.3.3. CS Exposure Induces Apoptosis in HaCaT Cells

**Figure 3:**
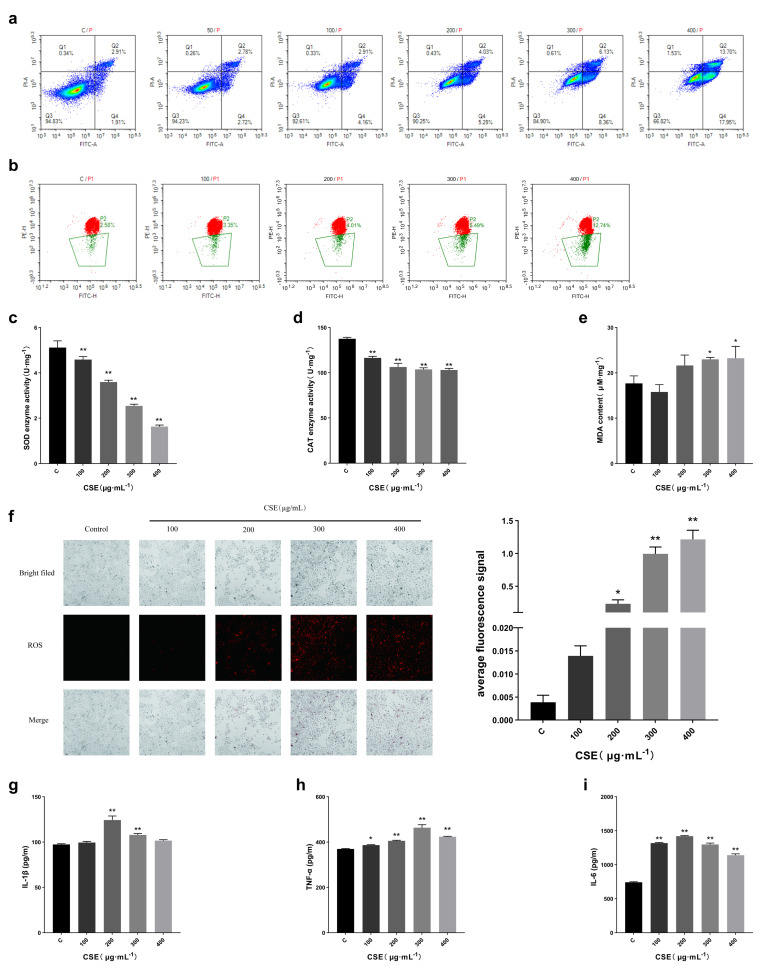
(**a**,**b**): CS exposure can induce apoptosis of HaCaT cells. (**a**). The results of apoptosis caused by different concentrations of CSE after 24 h. (**b**). The results of mitochondrial membrane potential after 24 h of exposure to different concentrations of CSE. c–f: CS exposure increases the level of oxidative stress in HaCaT cells, *n* = 3/group. (**c**). The results of SOD activity after 24 h of CSE treatment with different concentrations. (**d**). The effect on CAT enzyme activity after 24 h of exposure to different concentrations of CSE. (**e**). The effect of different concentrations of CSE on MDA content after 24 h. (**f**). The results of exposure to different concentrations of CSE for 24 h on ROS levels. (**g**–**i**): The effect of CS on the expression of inflammatory factors in HaCaT cells, *n* = 3/group. (**g**). The results of IL-1β levels after 6 h of CSE treatment with different concentrations. (**h**). The results of TNF-α levels after 6 h of 4 exposures to different concentrations of CSE. (**i**). Effects of different concentrations of CSE on IL-6 levels after 6 h. All data are displayed as mean ± SD. * *p* < 0.05, ** *p* < 0.01 (vs. control group).

Among the core targets of CS-induced skin damage, all targets were directly or indirectly involved in the process of cell apoptosis. GO/KEGG analysis also showed that apoptosis was involved. Therefore, flow cytometry was used to evaluate apoptosis and mitochondrial membrane potential. The results show that CS induces cell apoptosis and reduces mitochondrial membrane potential (Figure 3a,b).

#### 2.3.4. CS Exposure Induces Oxidative Stress in HaCaT Cells

The analysis of the core targets and GO/KEGG pathways of CS-induced skin damage showed that oxidative stress was also a key mechanism. Therefore, we detected markers related to oxidative stress, including SOD activity, CAT activity, MDA content and ROS level. The results showed that SOD and CAT activity were significantly reduced in HaCaT cells exposed to 100, 200, 300 and 400 µg/mL CSE (Figure 3c,d). The MDA content increased at 200, 300 and 400 µg/mL CSE concentrations and achieved statistical significance at 300 µg/mL CSE concentrations (Figure 3e). DHE fluorescence staining indicated that the ROS level in HaCaT cells exposed to different concentrations of CSE (100, 200, 300, 400 µg/mL) increased as compared to the control, of which the increase in the 200 µg/mL CSE group was statistically significant (Figure 3f). These results show that CS can induce oxidative stress in HaCaT cells, thus promoting skin damage.

#### 2.3.5. CS Exposure Induces Inflammatory Response in HaCaT Cells

In addition, the analysis also shows that the inflammatory reaction is the key to CS-induced skin damage. Therefore, we detected the inflammatory markers in the cell supernatant, including IL-1β, TNF-α and IL-6. The results show that after 6 h of cell exposure to CSE, the levels of IL-1β (Figure 3g), TNF-α (Figure 3h) and IL-6 (Figure 3i) increased and then decreased with the increase in CSE concentration. At 200 and 300 μg/mL CSE concentrations, the levels of IL-1β, TNF-α and IL-6 reached statistical significance. These results show a notable increase in IL-1β, TNF-α, and IL-6 levels after 6 h of moderate-concentration CSE intervention.

### 2.4. RT-qPCR Analysis

**Figure 4:**
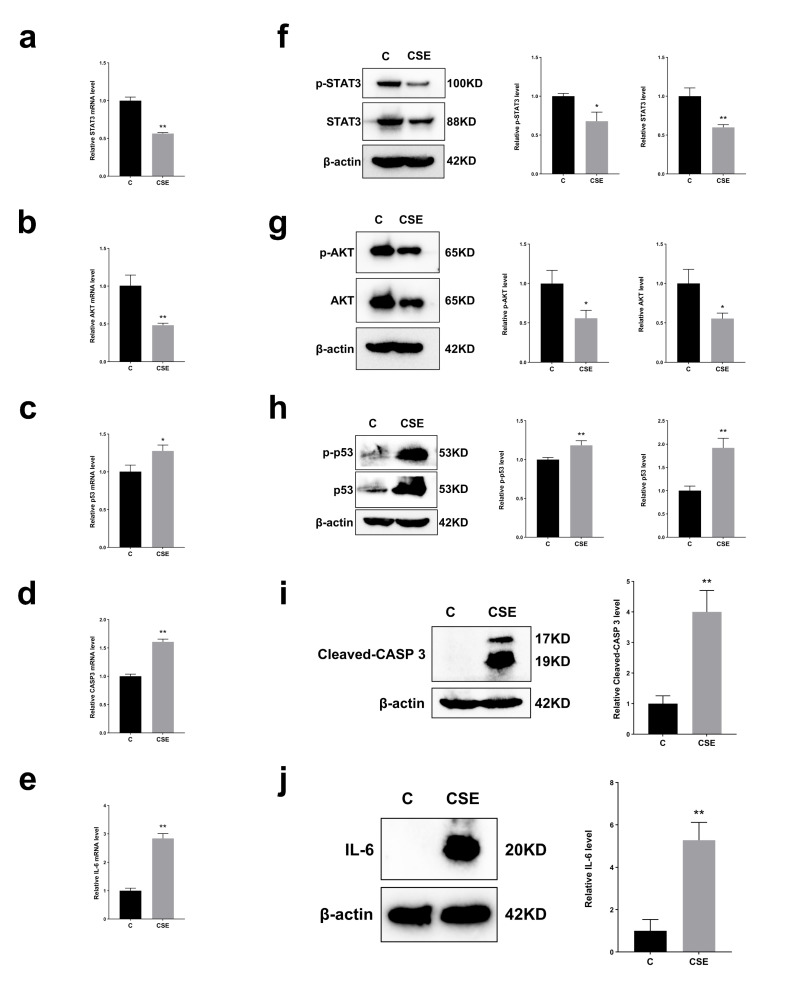
(**a**–**e**): Effect of CS on mRNA expression of key targets in HaCaT cells. We used β-actin as a reference, *n* = 3/group. (**a**). CS reduced STAT3 mRNA levels. (**b**). CS reduced AKT mRNA levels. (**c**). CS increased p53 mRNA levels. (**d**). CS increased CASP3 mRNA levels. (**e**). CS increased IL-6 mRNA levels. (**f**–**j**): Effect of CS on protein levels in HaCaT cells. We used β-actin as a reference, *n* = 3/group. (**f**). CS reduced p-STAT3 and STAT3 protein levels. (**g**). CS reduced p-AKT and AKT protein levels. (**h**). CS increased p-p53 and p53 protein levels. (**i**). CS increased Cleaved CASP3 protein levels. (**j**). CS increased IL-6 protein levels. All data are displayed as mean ± SD. * *p* < 0.05, ** *p* < 0.01 (vs. control group).

To study the expression of key targets in CS-induced skin injury, we measured mRNA levels of five major targets (STAT3, AKT1, TP53, CASP3, and IL-6) selected by PPI network analysis. The results of RT-qPCR showed that compared with the normal control group, mRNA expressions of STAT3 and AKT in cells exposed to 300 μg/mL CSE for 24 h decreased significantly (Figure 4a,b). On the contrary, p53, CASP3 and IL-6 expression levels experienced a considerable boost under the same conditions (Figure 4c,e). These results show that these core targets play a key role in CS-induced skin damage.

### 2.5. Western Blot Analysis

To study the core target and KEGG pathway of skin injury caused by CS exposure, we identified the key target and key proteins in related signaling pathways using WB analysis. The WB results showed that, compared to the normal control group, protein expression levels of p-STAT3, STAT3, p-AKT, and AKT were significantly downregulated in the CSE group exposed to 300 µg/mL CSE for 24 h (Figure 4f,g). Quantitative analysis of the p-STAT3/STAT3 and p-AKT/AKT ratios (Figure S2a,b) preliminarily indicated that CS may induce skin damage by inhibiting the PI3K-Akt and JAK-STAT signaling pathways. Conversely, protein expression levels of p-p53, p53, CASP3, and IL-6 were upregulated (Figure 4h–j), suggesting that CS might induce skin damage not only by activating the p53 pro-apoptotic pathway but also by engaging an inflammatory response.

### 2.6. Molecular Docking of CS Components with Key Targets

**Figure 5:**
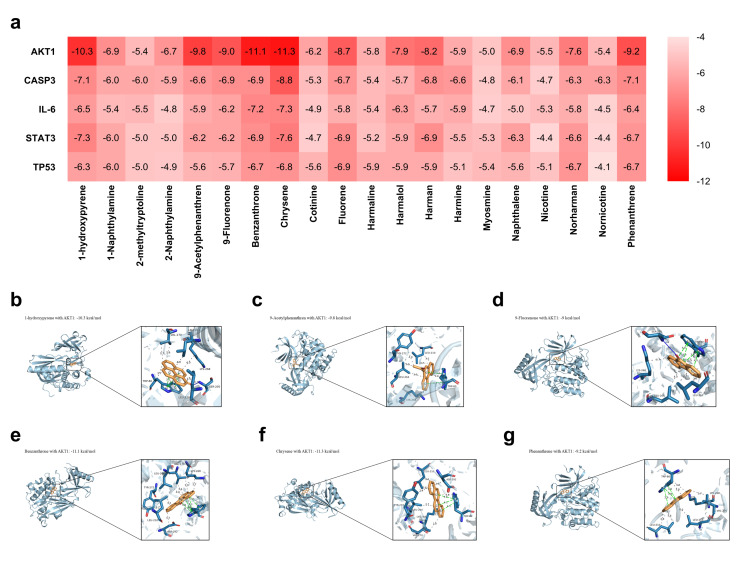
Molecular docking analysis of CS components and key targets related to skin damage. (**a**). A thermogram showing the binding affinity between five main targets and twenty CS components by docking calculation. (**b**–**g**): Six groups of molecular interaction diagrams with the strongest binding affinity. (**b**). AKT1 and 1-Hydropyrene, (**c**). AKT1 and 9-Acetylphenanthrene, (**d**). AKT1 and 9-fluoronone, (**e**). AKT1 and Benzanthrone, (**f**). AKT1 and Chrysene, and (**g**). AKT1 and Phenanthrene.

The molecular docking results showed that all five core targets could spontaneously bind to the 20 CS components, with binding energies below −4.1 kcal/mol (Figure 5a). The top six molecular interactions with the strongest binding affinity were selected for visualization: AKT1 with 1-hydroxypyrene, 9-Acetylphenanthrene, 9-Fluorenone, Benzanthrone, Chrysene, and Phenanthrene (Figure 5b–g).

## 3. Discussion

CS is a widespread environmental pollutant with complex chemical composition, which poses a persistent and serious threat to human health. The WHO reports that the global prevalence of smoking exceeds one billion individuals. Direct smoking or exposure to second-hand smoke causes up to 7 million deaths every year, posing a heavy burden on global public health [4,16,17]. In addition to causing serious indoor and outdoor air pollution, tobacco waste is also an important source of microplastics in the environment, causing continuous harm to soil and aquatic ecosystems [17]. CS contains more than 7000 compounds, many of which are toxic, mutagenic and carcinogenic [18]. These compounds can trigger a series of pathophysiological processes, including oxidative stress, inflammatory reactions, DNA damage, endothelial dysfunction and impaired immune function [19]. Given the complex chemical composition and well-documented systemic toxicity of CS, its impact on skin biology warrants mechanistic investigation.

This study integrates LC-MS analysis, network toxicology and in vitro experiments, and systematically studies the molecular mechanism of skin damage induced by CS exposure. The chemical characterization of CSE shows that alkaloids and PAHs (represented by nicotine and Phenanthrene) are the main pollutants. Preliminary calculations and evaluations indicate that several components, including Myosmine and 2-methyltryptoline, may be toxic to the skin. We used network toxicology to screen 57 potential targets for CS-induced skin damage and further identified the five core targets of STAT3, AKT1, TP53, CASP3 and IL-6 through PPI network analysis. Enrichment analysis using GO/KEGG indicated that signaling pathways, such as PI3K-Akt, p53, and JAK-STAT, are involved in the regulation of key biological processes. These predictions were further supported by subsequent in vitro experiments. Given that HaCaT cells, as the main component of the skin epidermis, are the first defense against environmental toxins, we chose them as the in vitro CS exposure model. Our findings indicated a marked decrease in STAT3 and AKT expression, accompanied by increased levels of p53, CASP3, and IL-6. Survival-promoting PI3K-AKT and JAK-STAT-related responses are suppressed, while p53-mediated apoptosis and IL-6-driven inflammation are activated. These alterations contribute to cytotoxicity and functional impairments in skin cells, manifesting as decreased cell metabolic activity, proliferative capacity and wound healing response and increased oxidative stress and inflammatory responses.

As the key “guardian” of the genome, p53 plays a central role in responding to cell stress signals (e.g., DNA damage) and determining cell fate (e.g., cell cycle blockage, aging or apoptosis) to maintain tissue homeostasis [20,21]. Our research shows that CS exposure significantly increases the level of oxidative stress in cells, accompanied by the upregulation of p53 transcription, total protein levels and p53 phosphorylation, indicating that the p53-related response is activated. This aligns with previous studies indicating that genotoxic stressors (e.g., ROS) in CS directly cause DNA damage, thereby activating p53 [22,23]. Activated p53, on the one hand, induces cell cycle arrest by regulating its downstream target p21, consistent with our observed decrease in cell proliferation capacity [24,25]. On the other hand, p53 can directly upregulate pro-apoptotic proteins (e.g., Puma and Bax), induce mitochondrial outer membrane permeabilization, subsequently activate the Caspase cascade (e.g., CASP3/CASP7), and ultimately execute the apoptosis program [24,26]. The findings of this study support this; that is, CS can decrease mitochondrial membrane potential, increase HaCaT cell apoptosis, and increase the expression of the CASP3 gene and protein.

This study found that in HaCaT cells exposed to CS, the gene expression, total protein content, and phosphorylated levels of STAT3 and AKT were significantly reduced on average, indicating that these survival responses are suppressed [27]. This impairment of the cellular defense system may contribute to the observed increase in LDH release, as well as the decreased cell metabolic activity and wound healing response. The dysfunction of these cells is closely related to the bad effects of a damaged skin barrier and slow wound healing [28,29]. Importantly, it has been reported that activated p53 can inhibit the functions of AKT and STAT3 by reducing transcription and other processes. Conversely, inhibited AKT and STAT3 can no longer restrict p53, forming a cycle that keeps p53 activated, which together determine whether a cell is alive or dead [30,31,32]. Our data are consistent with this research, which highlights the complex interaction between p53 and the AKT/STAT3 pathway in the skin toxicity caused by CS.

In addition, we observed a significant increase in IL-6 expression after CS exposure, highlighting the key role of the inflammatory response in its skin toxicity. Interestingly, despite the significant upregulation of IL-6, its classic downstream JAK-STAT signaling pathway has not been activated. Previous studies have shown that DNA-damaging agents can induce the expression of pro-inflammatory factors (e.g., IL-6) through the p53 pathway [33]. This suggests that the p53-mediated signaling pathway may dominate under CS exposure. At the same time, CS-induced oxidative stress is also an important trigger for pro-inflammatory transcription factors (e.g., IL-6). Inflammatory reactions will further aggravate oxidative stress, form a vicious circle of oxidative stress–inflammation, and synergize with the cell apoptosis pathway to accelerate skin cell damage [34].

Although this study provides preliminary insights into the molecular mechanisms underlying CS-induced skin damage, several limitations should be acknowledged. The composition of the CSE model is influenced by cigarette brand, preparation, and storage conditions, limiting its ability to fully recapitulate the complex mixture of gaseous and particulate pollutants encountered in real human exposure scenarios [35,36]. In addition, while network toxicology offers a useful predictive framework, its accuracy is constrained by the chemical complexity of CS and incomplete database coverage, and molecular docking alone is insufficient to identify the dominant bioactive constituents. Moreover, in vitro experiments were restricted to HaCaT cells, lacking the intact skin microenvironment, and molecular alterations observed under near-lethal injury conditions may be confounded by non-specific effects related to cell death. Finally, the analysis focused on selected time points and individual signaling components, rather than the dynamic behavior and full downstream output of entire pathways, and the translational relevance of these findings requires further validation in physiologically and clinically relevant models. Future studies should incorporate air–liquid interface exposure systems, 3D skin models, human skin tissues, and in vivo animal models to more accurately simulate cutaneous exposure to CS and enhance environmental and clinical relevance. The use of purified high-risk smoke constituents, combined with multi-time-point and multi-dose exposure designs, will facilitate systematic characterization of the transition from early stress responses to late-stage cellular damage. Furthermore, gain- and loss-of-function approaches based on genetic or pharmacological manipulation will be essential for establishing causal relationships. The pivotal role of NF-κB in inflammation regulation warrants further investigation into its underlying mechanisms. Finally, evaluating antioxidant and anti-inflammatory natural products or targeted therapeutics may provide mechanistic insight and potential intervention strategies to protect the skin health of smokers and individuals exposed to second-hand smoke.

## 4. Materials and Methods

### 4.1. Experimental Reagents

MTT was provided by GenXion (Guangzhou, China). We obtained a range of assay kits from Beyotime (Shanghai, China), including the LDH Cytotoxicity Assay, Annexin V-FITC Apoptosis Detection, JC-1 Mitochondrial Membrane Potential, Total Superoxide Dismutase with WST-8, Catalase Activity, Lipid Peroxidation MDA, 4% Paraformaldehyde, Crystal Violet, RIPA Lysis Buffer, and BeyoECL Star. The Dihydroethidium reagent was sourced from Solarbio (Beijing, China). The Pierce BCA Protein Assay Kit was acquired from Thermo Fisher Scientific (Waltham, MA, USA). JONLNBIO (Shanghai, China) supplied the ELISA kits for Human Interleukin 6 (IL-6), Human Tumor Necrosis Factor Alpha (TNF-α), and Human Interleukin 1 Beta (IL-1β). Protease and Phosphatase Inhibitor Cocktail Tablets were purchased from Roche (Basel, Switzerland). Antibodies targeting p-STAT3, STAT3, p-AKT, AKT, p-p53, p53, Cleaved Caspase 3, IL-6, and β-actin, along with Goat anti-rabbit IgG-HRP secondary antibodies, were obtained from Proteintech (Wuhan, China). Stripping Buffer was sourced from Cowin Biotech (Taizhou, China). We obtained kits, including the SteadyPure Universal RNA Extraction Kit II, Evo M-MLV RT Mix with gDNA Clean for qPCR Version 2, and the SYBR Green Premix Pro Taq HS qPCR Tracking Kit (Rox Plus), which were procured from Accurate Biology (Changsha, China).

### 4.2. Cell Culture

HaCaT cells were sourced from the National Collection of Authenticated Cell Cultures (NCOACC) (Shanghai, China). We nurtured these cells in DMEM medium (Gibco, Grand Island, NY, USA), supplemented with 10% fetal bovine serum (VivaCell, Shanghai, China) and 1% penicillin–streptomycin solution (Gibco, USA). The cells were maintained within a controlled incubator environment (37 °C, 5% CO_2_).

### 4.3. CSE Preparation and Characterization

The CSE was prepared using a modified Nakamura’s method [37]. To put it simply, we used a 25 mL syringe to pass 20 cigarettes (with filters) through 25 mL of 70% ethanol, in turn, under fixed conditions (aspirating for 10 s, stopping for 20 s). We repeated this method until all the cigarettes were used up. The obtained liquid was transferred to a centrifuge tube, dried with nitrogen, and then stored at −80 °C for later use. Before the experiment, the solid CSE was dissolved in DMSO and then mixed with the complete culture medium for dilution. A 0.22 μm filter was employed to sterilize subsequently.

For analysis, the prepared dry CSE was dissolved in 80% methanol at a concentration of 20 mg/mL and then sterilized with a 0.22 μm filter. The analysis was done on an Ultimate 3000 UPLC+-Q EX Active Focus LC-MS system (Thermo Fisher Scientific, Waltham, MA, USA), which was equipped with a Waters ACQUITY UPLC^®^ BEH C18 column (1.7 μm, 2.1 × 100 mm). The mobile phase featured 0.1% formic acid in water (A) and acetonitrile (B) with gradient elution: 0–1 min: 5% B; 1–7 min: 5–23% B; 7–10 min: 23–30% B; 10–15 min: 30–45% B; 15–30 min: 45–85% B; 30–32 min: 85–95% B; 32–36 min: 95% B; 36–37 min: 95–5% B; and 37–40 min: 5% B. We set the flow rate to 0.3 mL/min while keeping the column temperature steady at 35 °C and opted for a 1 μL injection volume. The HESI source was cranked up to 3.5 kV when running in positive-ion mode and dialed back to 3.2 kV for negative-ion mode. Data processing was performed using MSDIAL software (v5.5), compound identification was conducted by screening against the NIST mass spectral library, and percentage composition was calculated.

### 4.4. Network Toxicology Analysis

#### 4.4.1. CS Toxicity Analysis

The major components identified in CSE were selected. Their chemical structures, molecular information, and toxicological data came from PubChem (https://pubchem.ncbi.nlm.nih.gov, accessed on 26 August 2025) [14]. Skin toxicity assessments for these components, including skin irritation, corrosion potential, and sensitization risk, were performed using AttentiveSkin (https://bohrium.dp.tech/apps/attentive-skin, accessed on 28 August 2025), ADMETLAB 3.0 (https://admetlab3.scbdd.com, accessed on 28 August 2025), and Pred-Skin 3.0 (http://predskin.labmol.com.br/, accessed on 28 August 2025) platforms [15,38,39].

#### 4.4.2. Collection of CS and Skin Damage-Related Targets

Components assessed by AttentiveSkin, ADMETLAB 3.0, and Pred-Skin 3.0 as having skin irritation, corrosion potential, or sensitization risk were selected. The possible targets were accessed from Swiss Target Prediction (http://www.swisstargetprediction.ch/, accessed on 3 September 2025), SEA (https://sea.bkslab.org/, accessed on 3 September 2025), and CTD (https://ctdbase.org/, accessed on 3 September 2025) [40,41,42]. The screening criteria were as follows: Swiss Target Prediction: Probability > 0; SEA: Max TC ≥ 0.3 and *p* < 0.05; and CTD: Reference Count ≥ 2. We combined the targets from the three databases and removed duplicate parts to get the final set of CS-related targets.

Targets associated with skin damage were sourced from GeneCards (https://www.genecards.org/, accessed on 4 September 2025), TTD (http://bidd.group/group/cjttd/, accessed on 4 September 2025), and OMIM (https://omim.org/, accessed on 4 September 2025) using the keywords “skin damage”, “skin injury”, “skin lesion”, and “skin dysfunction”, with the species set to human [43,44]. For GeneCards, the top 200 genes ranked by relevance score related to skin damage were selected. The targets from the three databases were combined, the duplicates were removed, and the final target set related to skin damage was obtained.

#### 4.4.3. Screening of CS-Induced Skin Damage Targets and PPI Network Construction

Targets potentially linked to skin damage induced by CS were determined by intersecting CS-related targets with those associated with skin damage using an online Venn (https://bioinfogp.cnb.csic.es/tools/venny/, accessed on 4 September 2025), resulting in the creation of a Venn diagram [45]. The STRING database (https://string-db.org/, accessed on 5 September 2025) was used to import these intersection targets to construct a PPI network under the following conditions: Homo sapiens, high confidence (0.7), and FDR stringency (1%) [46]. The generated network was subsequently imported into Cytoscape (v3.10.0) for visualization. To identify functionally important nodes within the network, a topological analysis was performed using the CytoNCA plugin. Three commonly used network topology metrics were evaluated: degree centrality (DC), representing the number of direct interactions of a node; betweenness centrality (BC), reflecting the extent to which a node acts as a bridge within the network; and closeness centrality (CC), indicating the average distance between a node and all other nodes. Nodes with DC, BC, and CC values equal to or greater than the corresponding median values were retained through topological filtering and considered key targets for subsequent analyses.

#### 4.4.4. GO/KEGG Analysis

Using the DAVID database (https://davidbioinformatics.nih.gov/home.jsp, accessed on 5 September 2025), GO function annotation and KEGG pathway enrichment analyses were carried out on potential targets related to CS-induced skin damage [47]. The results were visualized using an online bioinformatics platform (https://www.bioinformatics.com.cn/, accessed on 5 September 2025).

### 4.5. Molecular Docking

We pulled the PDB files for core target proteins directly from RCSB (https://www.rcsb.org/, accessed on 6 September 2025), while the molecular structures of all 20 CS components in SDF format were sourced from PubChem (https://pubchem.ncbi.nlm.nih.gov, accessed on 6 September 2025) [14,48]. The core target proteins were identified based on *Homo sapiens*, X-ray diffraction, 1.5–3.0 A, crystal structures, and recent publications. The corresponding PDB IDs are as follows: AKT1 (pdb_00007nh4), CASP3 (pdb_00007rnc), IL-6 (pdb_00008ywq), STAT3 (pdb_00005ax3), and TP53 (pdb_00008wd2). Prior to docking, both protein and ligand structures underwent energy minimization. Proteins were pre-treated by removing water molecules and residual ligands, while ligands were minimized using the MM2 force field. Molecular docking analysis was conducted utilizing AutoDock Vina (v1.1.2), employing grid boxes covering the target protein’s binding site. The binding energies obtained from the docking process were used to assess the interaction strength between the core targets and the CS components. A binding energy < 0 kcal/mol indicates spontaneous binding, <−5 kcal/mol indicates good binding, and <−7 kcal/mol indicates strong binding. A lower binding energy suggests a stronger association and more stable binding between the component and the target. The docking results were presented as a heatmap, and the top six molecular interactions with strong binding affinity (such as hydrophobic interactions, hydrogen bonding, π-stacking (parallel)) were visualized using PLIP (https://plip-tool.biotec.tu-dresden.de/, accessed on 8 September 2025) and PyMOL (v3.0.3) [49].

### 4.6. Cell Experiments

#### 4.6.1. Detection of LDH Release, Cell Metabolic Activity, Proliferative Capacity and Wound Healing Response in HaCaT Cells

HaCaT cells were placed in 96-well or 6-well plates. After cell attachment, we added different concentrations of CSE to the culture.

**LDH Release:** We placed 10,000 cells per well in a 96-well plate and observed the release of LDH from HaCaT cells by CSE according to the instructions of the LDH Cytotoxicity Assay Kit. The absorbance was measured at 490 nm by a microplate reader (BioTek, Winooski, VT, USA), and then the percentage of LDH release was calculated by this formula: LDH release (%) = (OD_sample_ − OD_blank_)/(OD_total LDH release_ − OD_blank_) × 100%.

**MTT Assay:** We seeded 10,000 cells per well in 96-well plates and determined HaCaT cell viability post-CSE exposure using the MTT assay. Specifically, we added 0.5 mg/mL MTT, incubated at 37 °C in the dark for four hours, and measured the absorbance at 490 nm using a microplate reader (BioTek, Winooski, VT, USA). The cell survival rate was calculated by this formula:

Cell viability (%) = (OD_sample_ − OD_blank_)/(OD_control_ − OD_blank_) × 100%.

**Clonogenic Assay:** We placed 2000 cells per well in a 6-well plate. After 14 days of CSE exposure, we assessed the proliferation capacity of HaCaT cells. Specifically, following PBS washing, we added 1 mL of 4% paraformaldehyde for 20 min of fixation, stained with 1% crystal violet for 20 min, and then washed with PBS, dried, photographed, and recorded.

**Wound Healing Assay:** We placed 500,000 cells per well in a 6-well plate. When cell confluence reached 80%, we created a scratch wound and added different concentrations of CSE. We recorded the scratch wound under a microscope at 0 h and 24 h to investigate the effect of CSE exposure on HaCaT cell wound healing response.

#### 4.6.2. Detection of HaCaT Cell Apoptosis

A total of 300,000 HaCaT cells per well were seeded into 6-well plates. Following CSE exposure, the cells and their culture medium were collected. Then, the cells were mixed in Annexin V-FITC binding buffer, gently supplemented with 5 μL Annexin V-FITC and 10 μL propidium iodide solution, and incubated at room temperature in the dark for 20 min. Apoptosis rates were subsequently measured using a flow cytometer (Agilent, Santa Clara, CA, USA).

#### 4.6.3. Detection of Mitochondrial Membrane Potential in HaCaT Cells

A total of 300,000 HaCaT cells per well were seeded into 6-well plates. Cells exposed to CSE were harvested, incubated with prepared JC-1 staining solution at 37 °C in the dark for 20 min, and washed twice with JC-1 buffer. JC-1 accumulates within mitochondria under the influence of membrane potential. At high membrane potentials, it forms red fluorescent aggregates; at low membrane potentials, it remains as green fluorescent monomers. Mitochondrial membrane potential was subsequently measured using a flow cytometer (Agilent, Santa Clara, CA, USA) [50].

#### 4.6.4. Detection of SOD, CAT Activity, and MDA Levels in HaCaT Cells

We seeded 3 × 10^5^ HaCaT cells per well in a 6-well plate. Cell lysates treated with CSE were collected, and the supernatants were separated. The total protein concentration in the samples was determined using the BCA method. Subsequently, reagents were prepared according to the kit instructions to measure SOD, CAT, and MDA levels. A microplate reader (BioTek, Winooski, VT, USA) was employed to measure SOD absorbance at 450 nm, CAT absorbance at 520 nm, and MDA absorbance at 532 nm.

#### 4.6.5. Detection of ROS Level in HaCaT Cells

HaCaT cells were spread in 6-well plates at a density of 300,000 cells per well. Cells exposed to CSE were incubated in the prepared DHE staining solution at 37 °C in the dark for 20 min. Following PBS washing, red fluorescence signals were measured and photographed using an inverted fluorescence microscope (Nikon, Tokyo, Japan).

#### 4.6.6. Detection of IL-1β, TNF-α, and IL-6 Levels in HaCaT Cells

HaCaT cells were planted in 6-well plates with 300,000 cells in each well. We collected cell supernatants following exposure to CSE. The levels of IL-1β, TNF-α, and IL-6 were measured according to the kit protocols, with absorbance at 450 nm measured using a microplate reader (BioTek, Winooski, VT, USA).

### 4.7. Quantitative Real-Time PCR (RT-qPCR)

We collected RNA from HaCaT cells exposed to 300 μg/mL CSE for 24 h using an RNA extraction kit. cDNA was then synthesized using the Evo M-MLV RT Kit under the following conditions: 37 °C for 15 min, followed by 85 °C for 5 s. Subsequent RT-qPCR analysis was performed using the SYBR Green Premix Pro Taq HS qPCR Kit under the following conditions: 95 °C for 30 s, followed by one cycle; 95 °C for 5 s and 60 °C for 30 s, repeated for 40 cycles. β-actin was employed as the internal reference gene. Primer sequences were custom-designed by Accurate Biology (Changsha, China), as detailed in Table S2.

### 4.8. Western Blot

We lysed HaCaT cells exposed to 300 μg/mL CSE for 24 h using RIPA lysis buffer (containing mixed surfactants, including non-ionic, ionic and deoxycholate salts, supplemented with protease inhibitors and phosphatase inhibitors) and an ultrasonic homogenizer (set at 30% intensity, total 5 min, operating for 10 s with 10-s intervals) [51]. The resulting lysate was centrifuged (4 °C, 12,000 rpm, 5 min), and the supernatant was collected. The protein concentration was determined using the BCA assay. The supernatant was mixed with 1× loading buffer and heated to 100 °C in a metal bath to denature proteins. Samples were then separated by SDS-PAGE and transferred onto PVDF membranes. The membranes were blocked with 5% skimmed milk for one hour. Subsequent incubation was performed with the following primary antibodies: p-STAT3 (1:2000), STAT3 (1:8000), p-AKT (1:2500), AKT (1:5000), p53 (1:5000), p53 (1:25,000), cleaved CASP3 (1:1000), IL-6 (1:1000), and β-actin (1:5000) overnight at 4 °C. The following day, incubation was performed for one hour with goat anti-rabbit IgG-HRP secondary antibody (1:2000). For proteins of similar molecular weight, the membranes were stripped using stripping buffer, re-blocked, and re-incubated. The strips were captured using ECL reagents in a chemiluminescent imaging system (Bio-Rad, Hercules, CA, USA) and analyzed with Image Lab software (v6.1).

### 4.9. Statistical Analysis 

Data are presented as mean ± SD, with significance determined by GraphPad Prism (v10.1.2) (*p* < 0.05).

## 5. Conclusions

In conclusion, this study proposes an integrated toxicological framework through which CS exposure compromises skin cell homeostasis, characterized by the suppression of survival-associated signaling and the activation of apoptotic and inflammatory responses. By combining LC–MS-based chemical profiling, network toxicology, molecular docking, and cellular validation, we suggest that inhibition of the PI3K–AKT- and JAK–STAT-related responses, together with enhanced p53-mediated apoptosis and IL-6-associated inflammation, represents a central mechanism underlying CS-induced skin damage. Importantly, this integrative strategy not only strengthens the biological interpretation of in vitro toxicological data but also provides a systematic approach for identifying key molecular vulnerabilities in complex environmental exposures. These findings offer mechanistic insight into the potential risks of cigarette smoke to skin health and may inform the development of targeted protective or preventive strategies in the context of tobacco-related environmental exposure.

## Figures and Tables

**Table 1:** Representative high-abundance alkaloids and PAHs identified in CSE.

NO.	Compound Name	Formula	RT (min)	Precursor *m*/*z*	Reference *m*/*z*	Adduct	Content (%)
**Alkaloids**							
1	Nicotine	C_10_H_14_N_2_	1.18	163.12	163.12	[M+H]+	5.1946%
2	Norharman	C_11_H_8_N_2_	6.68	169.08	169.08	[M+H]+	0.7365%
3	Harman	C_12_H_10_N_2_	7.38	183.09	183.09	[M+H]+	0.3958%
4	Myosmine	C_9_H_10_N_2_	2.67	147.09	147.09	[M+H]+	0.2791%
5	Cotinine	C_10_H_12_N_2_O	1.28	177.10	177.10	[M+H]+	0.2303%
6	Harmaline	C_13_H_14_N_2_O	5.45	215.12	215.12	[M+H]+	0.2126%
7	2-methyltryptoline	C_12_H_14_N_2_	9.03	187.12	187.12	[M+H]+	0.1840%
8	Harmalol	C_12_H_12_N_2_O	12.82	201.10	201.10	[M+H]+	0.1571%
9	Nornicotine	C_9_H_12_N_2_	4.67	149.11	149.11	[M+H]+	0.0962%
10	Harmine	C_13_H_12_N_2_O	4.36	213.10	213.10	[M+H]+	0.0792%
**Polycyclic Aromatic Hydrocarbons**							
1	Phenanthrene	C_14_H_10_	5.64	178.09	178.08	[M]+	0.0463%
2	2-Naphthylamine	C_10_H_9_N	4.73	144.08	144.08	[M+H]+	0.0444%
3	Fluorene	C_13_H_10_	2.07	167.08	167.09	[M+H]+	0.0403%
4	Naphthalene	C_10_H_8_	1.29	128.07	128.06	[M]+	0.0361%
5	1-Naphthylamine	C_10_H_9_N	3.86	144.08	144.08	[M+H]+	0.0228%
6	9-Fluorenone	C_13_H_8_O	13.93	181.06	181.06	[M+H]+	0.0140%
7	1-hydroxypyrene	C_16_H_10_O	4.75	217.07	217.07	[M-H]-	0.0022%
8	Chrysene	C_18_H_12_	4.58	229.10	229.10	[M+H]+	0.0021%
9	9-Acetylphenanthren	C_16_H_12_O	18.56	221.10	221.10	[M+H]+	0.0021%
10	Benzanthrone	C_17_H_10_O	1.42	231.08	231.08	[M+H]+	0.0020%

Table 1 lists selected alkaloids and polycyclic aromatic hydrocarbons identified by LC-MS analysis, exhibiting relatively high relative abundance and clear toxicological relevance; other detected constituents present in the complete extract are not listed individually.

**Table 2:** Molecular formula, SMILES, and skin toxicity of CS.

	Molecular Formula		AttentiveSkin		ADMETLAB 3.0	Pred-Skin 3.0
Name		SMILES	Irritating *	Corrosive #	Sensitization	Sensitization
Nicotine	C_10_H_14_N_2_	CN1CCC[C@H]1C2=CN=CC=C2	90.17%	99.94%	0.869	−
Norharman	C_11_H_8_N_2_	C1=CC=C2C(=C1)C3=C(N2)C=NC=C3	80.49%	20.52%	0.687	+
Harman	C_12_H_10_N_2_	CC1=NC=CC2=C1NC3=CC=CC=C23	60.65%	7.53%	0.693	−
Myosmine	C_9_H_10_N_2_	C1CC(=NC1)C2=CN=CC=C2	92.08%	99.96%	0.959	+
Cotinine	C_10_H_12_N_2_O	CN1[C@@H](CCC1=O)C2=CN=CC=C2	76.18%	99.73%	0.771	−
Harmaline	C_13_H_14_N_2_O	CC1=NCCC2=C1NC3=C2C=CC(=C3)OC	17.40%	8.31%	0.333	+
2-methyltryptoline	C_12_H_14_N_2_	CN1CCC2=C(C1)NC3=CC=CC=C23	54.34%	93.32%	0.856	+
Harmalol	C_12_H_12_N_2_O	CC1=NCCC2=C1NC3=C2C=CC(=C3)O	39.08%	10.66%	0.736	+
Nornicotine	C_9_H_12_N_2_	C1C[C@H](NC1)C2=CN=CC=C2	91.00%	99.93%	0.865	−
Harmine	C_13_H_12_N_2_O	CC1=NC=CC2=C1NC3=C2C=CC(=C3)OC	20.85%	0.58%	0.273	+
Phenanthrene	C_14_H_10_	C1=CC=C2C(=C1)C=CC3=CC=CC=C32	94.81%	1.11%	0.607	+
2-Naphthylamine	C_10_H_9_N	C1=CC=C2C=C(C=CC2=C1)N	93.12%	49.55%	0.606	+
Fluorene	C_13_H_10_	C1C2=CC=CC=C2C3=CC=CC=C31	93.75%	0.77%	0.793	+
Naphthalene	C_10_H_8_	C1=CC=C2C=CC=CC2=C1	97.34%	1.81%	0.678	+
1-Naphthylamine	C_10_H_9_N	C1=CC=C2C(=C1)C=CC=C2N	93.03%	48.68%	0.776	+
9-Fluorenone	C_13_H_8_O	C1=CC=C2C(=C1)C3=CC=CC=C3C2=O	89.41%	0.80%	0.727	+
1-hydroxypyrene	C_16_H_10_O	C1=CC2=C3C(=C1)C=CC4=C(C=CC(=C43)C=C2)O	85.30%	30.33%	0.906	+
Chrysene	C_18_H_12_	C1=CC=C2C(=C1)C=CC3=C2C=CC4=CC=CC=C43	89.46%	0.61%	0.333	+
9-Acetylphenanthren	C_16_H_12_O	CC(=O)C1=CC2=CC=CC=C2C3=CC=CC=C31	80.17%	0.86%	0.566	−
Benzanthrone	C_17_H_10_O	C1=CC=C2C(=C1)C3=CC=CC4=C3C(=CC=C4)C2=O	77.71%	0.37%	0.865	+

* According to the GHS standard, irritation denotes reversible damage to the skin, ^#^ whilst corrosivity refers to irreversible damage to the skin [15].

## Data Availability

Data will be made available on request.

## Supplementary Materials

The following supporting information can be downloaded at: https://www.mdpi.com/article/10.3390/ijms27052392/s1.

## Abbreviations

The following abbreviations are used in this manuscript:

p-STAT3	Phosphorylated Signal Transducer and Activator of Transcription 3
STAT3	Signal Transducer and Activator of Transcription 3
p-AKT	Phosphorylated Protein Kinase B
AKT	Protein Kinase B
p-p53	Phosphorylated p53 Protein
p53	Cellular Tumor Antigen p53
MTT	3-(4,5-Dimethylthiazol-2-yl)-2,5-diphenyltetrazolium bromide
SOD	Superoxide Dismutase
CAT	Catalase
MDA	Malondialdehyde
ROS	Reactive Oxygen Species
LC-MS	Liquid Chromatography–Mass Spectrometry
