# Supplementary material for: Unraveling the Molecular Mechanisms Linking Cigarette Smoke Exposure to Skin Damage
Source: Int J Mol Sci. 2026 Mar 4;27(5):2392. doi: 10.3390/ijms27052392 (PMC12985281; doi:10.3390/ijms27052392)
Supplement: Supplementary file 1 [file ijms-27-02392-s001.zip › Supplementary figure 1.pdf]

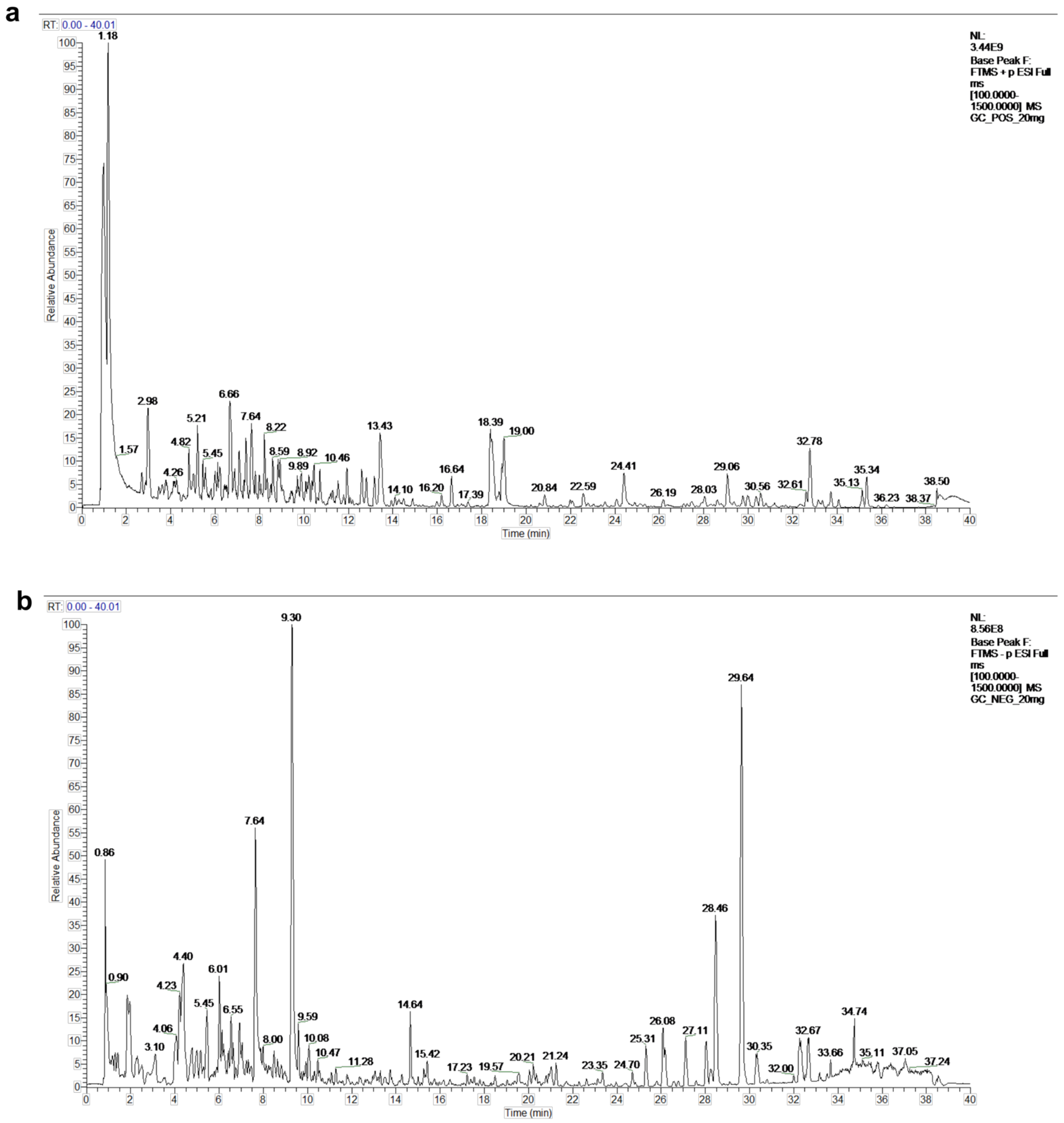

Fig. S1. TIC in positive ion mode and negative ion mode. a. TIC data obtained by QE mass spectrometer in positive ion mode. b. TIC data obtained by QE mass spectrometer in negative ion mode.
