# Supplementary material for: Unraveling the Molecular Mechanisms Linking Cigarette Smoke Exposure to Skin Damage
Source: Int J Mol Sci. 2026 Mar 4;27(5):2392. doi: 10.3390/ijms27052392 (PMC12985281; doi:10.3390/ijms27052392)
Supplement: Supplementary file 1 [file ijms-27-02392-s001.zip › Supplementary figure 2.pdf]

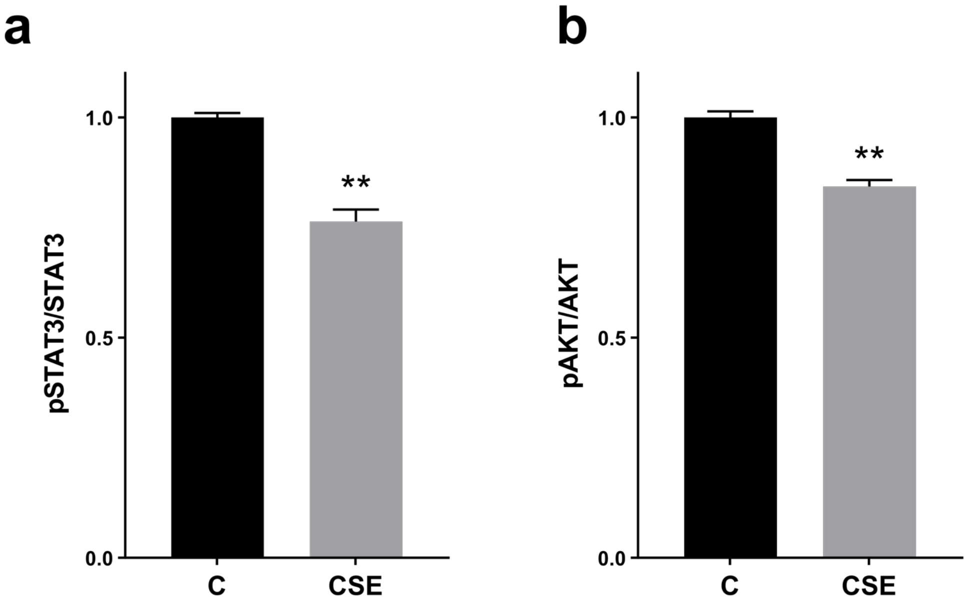

Fig. S2. a. Quantitative analysis of the p-STAT3/STAT3 ratio. b. Quantitative analysis of the p-AKT/AKT ratio. n = 3/group.
